# Clinical Holistic Medicine: Social Problems Disguised as Illness

**DOI:** 10.1100/tsw.2004.27

**Published:** 2004-05-11

**Authors:** Søren Ventegodt, Mohammed Morad, Isack Kandel, Joav Merrick

**Affiliations:** ^1^ The Quality of Life Research Center, Teglgårdstræde 4-8, DK-1452 Copenhagen K, Denmark; ^2^ Division of Community Health, Ben Gurion University, Beer-Sheva, Israel; ^3^ Faculty of Social Science, Department of Behavioral Sciences, Academic College of Judea and Samaria, Ariel, Israel; ^4^ National Institute of Child Health and Human Development, Office of the Medical Director, Division for Mental Retardation, Ministry of Social Affairs, Jerusalem and Zusman Child Development Center, Division of Pediatrics and Community Health, Ben Gurion University, Beer-Sheva, Israel

**Keywords:** quality of life, QOL, philosophy, human development, holistic medicine, public health, holistic health, holistic process theory, life mission theory, group therapy, Denmark

## Abstract

Many of the diseases seen in the clinic are actually symptoms of social problems. It is often easier for the physician to treat the symptoms than to be a coach and help the patient to assume responsibility in order to improve quality of life, social situation, and relations. If the physician ignores the signs of the disease as a symptom of social problems, and treats the patient with pharmaceuticals, he can give the patient the best justification in the world not to do anything about the situation. It is very important that the physician is not tricked by the games the socially troubled patient, more or less unconsciously, is playing. A firm and wise attitude that confronts the patient with his or her lack of responsibility for solving social problems seems to be a constructive way out. The physician can give holding and support, but the responsibility must remain with the patient. Often it is better for the patient that the physician abstains from giving drugs that can remedy the symptoms and takes the role of a coach instead. Suffering is not necessarily bad, suffering is actually highly motivating and often the most efficient source of learning. Coaching can help the patient canalize his motivation into highly constructive considerations and behavior. A holistic approach thus gives the patient learning and helps him rehabilitate his social reality. Concerning children with recurrent or chronic pain, we have observed an overuse of painkillers, where we believe part is of a psychosomatic nature due to poor thriving in the family. Here the physician has an important job helping the parents to develop as persons, teaching them the basic holding of awareness, respect, care, acknowledgment and acceptance of their child. Most of the chronic pain and discomfort with children can be improved if the physician understands how to use the holistic medical toolbox.

## INTRODUCTION

There are some people that cannot be helped by their physician because their symptoms are more or less direct manifestations of social difficulties. Physicians, especially in the U.S., have thus been criticized for treating social problems by prescribing pharmaceuticals for the symptomatic physical complaints [1]. Results from a logistic regression analysis showed that in women (but not in men), problems in the relationship with spouse or partner increased the probability of being a general practice attendee more than twofold[2]. It is well known from many studies that social and medical problems are strongly connected[3], and it is also known that the quality of the patients' social relationships are highly correlated to self-perceived mental and physical health[4,5]. In our experience, many symptoms connected to social problems are somatizations that will vanish into thin air once the difficulties are overcome. It may, however, be difficult to get that far, particularly if the patients believe that they are ill and use illness as an excuse to avoid solving their social and domestic problems.

Excuses are very common because it is difficult to take responsibility for all aspects of our lives. Somatic or mental illness can be a marvelous excuse because everybody accepts it immediately. But to a physician, the situation is more difficult to accept when the patient really has no somatic illness. The patient supposedly has terrible back pain, but walks into the room completely unaffected, sits down, and speaks in a lively manner. Then, suddenly, there is a shift, and all the pain in the back appears, as if by order. It is hard not to smile, but it may be said in the patient's defense that often he or she is only partially aware of what is going on. Talking about the pain can work miracles, if the patient is prepared to improve his or her life. Before we look at examples of social problems disguised as disease, let us look at how life in general can be improved.

## THE BASIS FOR CLINICAL HOLISTIC MEDICINE

The life mission theory[6,7,8,9,10,11] states that everybody has a purpose of life or has a huge talent. Happiness comes from living this purpose and succeeding in expressing the core talent in your life. To do this, it is important to develop as a person into what is known as the natural condition, a condition where you know yourself and use all your efforts to achieve what is most important for you. The holistic process theory of healing[12,13,14,15] and the related quality of life theories[16,17,18] state that the return to the natural state of being is possible whenever the person gets the resources needed for existential healing. The resources needed are, according to the theory, holding in the dimensions: awareness, respect, care, acknowledgment and acceptance with support and processing in the dimensions: feeling, understanding and letting go of negative attitudes and beliefs. The precondition for holistic healing to take place is trust and the intention for the healing to take place. Existential healing is not a local healing of any tissue, but a healing of the wholeness of the person, making him much more resourceful, loving, and knowledgeable of himself and his own needs and wishes. In letting go of negative attitudes and beliefs, the person returns to a more responsible existential position and an improved quality of life. The philosophical change of the person healing is often a change towards preferring difficult problems and challenges, instead of avoiding difficulties in life[19,20,21,22,23,24,25,26]. The person who becomes happier and more resourceful often also becomes more healthy, talented, and more able to function[27,28,29].

## EXAMPLES OF SOCIAL PROBLEMS DISGUISED AS DISEASE

We believe that the best way to illustrate our thoughts and intentions is to use case stories. The following case reports will therefore illustrate the problem from different angles.

> **Girl, aged one year, who is constantly ill**: Frequently ill, almost constantly since January. Violent pain makes her throw herself back and forth, uncontrollable. Examination: Speech development normal, knows only a few words, somewhat poor psychomotor development, walks unsteadily and often falls and hurts herself. Otoscopy: Red tympanic membrane bilaterally /Otitis media/ Prescribe penicillin. Socially: Her mother visits a crisis centre. They have found a family for respite care. The situation seems to be very hard for the patient.

This is an example of the extreme situation where the correlation between somatic illness and social circumstances is spelled out. Indeed, that is how young physicians learn it: first we understand the rough cases and then gradually the more subtle ones. And finally we are able to see the correlation, which nobody else can see, if we keep looking for it with ever more profound and subtle techniques.

> **Girl, aged seven years, with back problems:** Seven-year-old girl comes together with her father. She has low cervical back pain, projecting spinous process on palpation. Headache. Examination: Tension in the long back muscles. Her father says that he has no energy left for her, when he gets home, has major problems at work, will probably lose his job soon and is looking for a new one. Conversation about children's needs. There is not much to be done except to offer the father another conversation about better daytime planning. However, stretching the back helps the patient.

Suppressed feelings go straight into the back, in both children and adults. Often, it is difficult to change the situation for the adult. Back problems in children and adolescents with no direct traumatic cause or objective finding should alert the physician to look for psychosocial causes.

> **Girl, aged 14 years, with abdominal pain:** Over the past year several episodes with severe abdominal pain. Examination: Normal examination. During the conversation it is revealed that her parents have just separated. She has problems at school and also feels that she has to take too much responsibility for her two sisters, because her mother seems to be out of strength and very short tempered. /Psychosomatic abdominal pain/ We agree that she should discuss the situation with the mother and return for a conversation together.

Abdominal pain is a common childhood symptom that can range from mild discomfort to a life-threatening emergency requiring immediate attention. In most cases, surgery will not be required, but children with such symptoms should have a thorough checkup to make sure there is not a serious underlying problem. It can be difficult to diagnose the cause of abdominal pain, because many conditions affecting various parts of the body can produce abdominal pain. In infancy, the most common cause of abdominal pain is colic. As the child grows older, abdominal pain may be associated with minor disruptions of normal body functions (such as constipation) or with a variety of organic disorders or emotional problems. Often the pain accompanies diseases of the abdominal organs, such as the intestines, liver, pancreas, and stomach. But it may be relayed from other, more distant parts of the body; pneumonia and tonsillitis (strep throat), for example, sometimes cause abdominal symptoms.

The term recurrent abdominal pain (RAP) has been used and defined in various ways over time, but begins with a reference to Apley's criteria[30]. RAP is characterized by three or more episodes of abdominal pain that occur over at least 3 months and are severe enough to interfere with activities, such as school attendance and performance, social activities, and participation in sports and extracurricular activities. Clinically, these episodes are characterized by vague abdominal pain that may be dull or crampy, lasts for less than 1 h, and poorly localized or periumbilical. The pain frequently presents with nausea, vomiting, and other signs of autonomic arousal. Though the term RAP is most often used to refer to functional abdominal pain, Apley's original description is broad and does not have specific etiological implications. The majority of children with RAP do not have a specific physical disorder or organic disease. Most investigators report that only 5–10% of affected children have an organic cause for their pain.

Probably the hardest thing for the child is the need to be loyal to both parents and this can easily lead to a conflict that cannot be resolved. There is a deep psychological explanation for this need for two-sided loyalty, namely that each individual contains the two genders, represented by our parents. The quality of our future love life depends on how lovingly these two aspects of ourselves meet.

> **Male, aged 26 years, with back pain:** The patient asks for a medical certificate, but there is absolutely nothing wrong with his back, and pain cannot be provoked in any way. No tenderness, no spinal anomalies, normal flexion forwards and sideways. He has been off work for three months and brings papers that should have been filled out long ago.

If we are entirely unable to provoke the back pain claimed by the patient, we quite simply do not believe it exists. We know about back pain, we know how it hurts, and what makes it hurt. In addition, we can often tell when patients are lying, which does not make it any easier to believe in their stories. And sometimes, when the motive for lying is obvious — typically, as in this case, social problems at the workplace that the patient has been unable to solve — it may be better to grab the bull by its horns rather than allow a downward path, which a long-term sick leave often is. In this situation, as physicians we cannot help him; he has to find another job.

> **Female, aged 29, Separation:** Comes in order to solve the problems relating to separation from her former husband. She has two daughters aged 5 and 7 years. It is suggested to her that they spare the children their mutual problems and make the following arrangement: that the children live with their mother and stay with their father every second weekend from end of school on Fridays to start of school on Mondays (the children to be handed over to the other parent after school) and every Wednesday from end of school until start of school on Thursdays (the children to be handed over to the other parent after school), and that they share all holidays equally between them so that the father spends the first half of the holidays with the children, and the mother the second half. This arrangement should continue until the children are 12 years old, whereupon they should decide themselves where they want to live. Generally, it is working much better now, and the patient seems to be getting the situation under control. She has withdrawn her report of violence by her former husband.

We must often help to solve social problems and often experience that the symptoms with which the patient present — usually not the social problems, but physical or mental disorders — disappear when everything else falls into place. For the patient, it is of extreme value that the physician will take a holistic approach and help the patient take a comprehensive view of reality.

> **Male, aged 30 years, with neck pain:** Becomes dizzy **—** especially when smoking **—** has had severe neck pain, difficulty in bending his head to his chest. Currently unemployed, his wife gives him a massage in the tense regions. Does not want ibuprofen [NSAID] or similar medication. Prescribe stretching of antagonistic muscles, which proves effective. Should return in two weeks for new treatment, if the problem has not been solved by then.

“Unemployment” often covers a range of problems such as low self-esteem, social marginalization, low functional capacity, poor health, and low quality of life. People who are unemployed for the long term often do not just need a job; they need a rehabilitation program. However, it is very convenient to blame all problems on unemployment. The truth is that any person who has a high personal energy level, well-developed enjoyment of life, and a minimum of direction in life will never remain unemployed for very long without finding something interesting to do, pay or no pay. We do not condemn the unemployed, we merely encourage them, if they are receptive, to address the real problem — low functional capacity — and then to do something about it. Life skills can be developed, if there is a will to do so.

> **Male, aged 32 years, with back pain:** Back pain, on sick leave. No physical injury of the back, only severe muscle tension corresponding to the lower back. Manipulated with good effect. Patient claims to be incapacitated; no apparent evidence to that effect. He is put on sick leave for another week with follow-up.

This patient claims to have back pain, but in the opinion of the physician, there is nothing wrong with him. He has some muscle tension, but does not even appear to be in much pain. His gait is relaxed and quite natural, and he moves freely. In our opinion, muscle tension is not a disease, but rather something all of us experience because we unconsciously hide away our difficult feelings in the muscles. Back and low back muscles, in particular, are suitable for storing acute problems. But this patient is hoping to be put on long-term sick leave. General medical practice requires us to grant him another week to get better.

He is annoyed that the physician does not “buy” his claims of terrible pain. He claims that the physician cannot possibly know the kind of pain he is in. But we have a different opinion, namely that a good physician knows very well how much pain a patient is in. He also knows how easy or difficult it is to tolerate the pain. We believe that a physician with empathy knows a lot about his patients’ subjective condition, although naturally he cannot be absolutely certain.

> **Female, aged 57 years, with pain everywhere:** Complains of pain in the head, arms and hands, shoulders, upper and lower back, buttocks, knees and feet. Muscle pain all over the body on physical examination. Very tense. Recommended to swim twice a week. It is important that the patient uses her body and learns to relax at the same time. Interpreter and language problems. The problem is probably also cultural, since she does not speak Danish. Panodil [paracetamol] two tablets as required when she is active. Stronger medication needs to be considered. A language course and an integration programme would also benefit the patient, but that is hardly realistic due to low motivation.

Living in Denmark without knowing the language is not pleasant. Many foreigners never learn to speak Danish resulting in social isolation, feeling of being dispensable, and unhappiness may often lead to somatization and incurable pain.

> **Male, aged 79 years, with loneliness:** Home visit. He telephones complaining of gasping for air. On my arrival he is quite unaffected, has been feeling scared and lonely, he says, and presumably his inhalation spray may be empty. Would like some more cough mixture. The patient is calmed down and invited to return in two weeks to go through it all. Prescription: continue with Berodual [ipratropium, phenoterol], cough mixture.

Biomedicine helps elderly and weak people to survive and lead a decent and reasonably symptom-free life. Nevertheless, people decline, their general condition deteriorates, they function less and less well, they become old, isolated and lonely, until death catches up with them. Is that natural? However natural death is, we find the decline, the loneliness, and social exclusion equally unnatural, this constant loss of altitude until one cannot spread one’s wings at all.

## DISCUSSION

Many of the diseases we identify, diagnose, and treat with biomedicine might actually be mere symptoms of social conditions that weaken the patient's whole organism or simply somatize. If a social problem is treated like a disease, it is likely that the patient will not fight as eagerly as if he or she would if told that this condition was caused by actual problematic social circumstances. As physicians, we want to be merciful and helpful, but by treating symptoms and not confronting the patient with our understanding of the whole situation, we will not be true to our profession. If we are unable to point out the causal relations between poor living and poor quality of life, or subjective and objective symptoms of disease, we might obtain just the opposite effect. We can this way make the patient a passive victim to a nonexistent disease, or a health condition that actually could be alleviated, at least with some likelihood, by the patient him- or herself.

Much of our dissatisfaction with biomedicine is related to the fact that only rarely is it able to restore health, enjoyment of life, vitality, vital energy, and social worth in chronic patients, i.e., in the majority of the population over the age of 45 years of age.

As the many cases in this paper have demonstrated clearly, social problems are not difficult to alleviate when the physician has a clear understanding and insight. Concerning children, many of the problems we see in the clinic might be symptoms of familiar dysfunction or stress. Psychosomatic symptoms are, by definition, clinical symptoms with no underlying organic pathology. Common symptoms seen in children include abdominal pain, headaches, enuresis, chest pain, fatigue, limb pain, back pain, or worry about health and difficulty breathing. The prevalence of psychosomatic complaints in children and adolescents has been reported to be between 10 and 25%[31]. Potential sources of stress in children and adolescents include schoolwork, family problems, peer pressure, chronic disease or disability in parents, family moves, psychiatric disorder in parents, and poor coping abilities. Characteristics that favor psychosomatic basis for symptoms include vagueness of symptoms, varying intensity, inconsistent nature and pattern of symptoms, presence of multiple symptoms at the same time, chronic course with apparent good health, delay in seeking medical care, and lack of concern on the part of the patient. A thorough medical and psychosocial history and physical examination are the most valuable aspects of diagnostic evaluation in order to rule out organic etiology.

Sometimes the parents do not understand the character of the child or do not acknowledge its talents and purpose of life, what we a little poetically like to call "the essence of its soul". In this case, personal development is needed for the parents if the child is to heal existentially[7,10,14]. This can be brought around if the physician works with open eyes and a warm heart for his patients. We therefore call for a new medical approach that develops people and supports them in their effort to live long, good, and purposeful lives. Often, biomedicine only helps people survive the acute crises resulting in continued chronic decline. By contrast, it has little to offer in relation to the ordinary course of life of people in the Western world with ever decreasing quality of life, health, and functional capacity. We believe that using the toolboxes of the manual and consciousness-based medicine actually can empower the physician to help his patient more, even with problems related to existential issues like love, respect, sexuality, social functioning, and general attitudes towards life and other.

## CONCLUSION

Many of the diseases seen in the general practitioner clinic are mere symptoms of social problems. It is often easier for the physician to treat the symptoms than it is to help the patient assume responsibility and improve his or her quality of life, social situation, and relations.

If the physician ignores that the signs of a disease could be symptoms of social problems, he can give the patient the best justification in the world not to do anything about the situation. It is very important that the physician is not tricked by the games the socially troubled patient can play, more or less unconsciously. A firm and wise attitude that confronts the patient with his or her lack of responsibility for solving the social problems seems to be the only constructive way out of the situation. The physician can give holding and support, but the responsibility must remain with the patient. Often it is better for the patient that the physician abstains from giving drugs that can remedy the symptoms and takes the role of a coach instead. Suffering is not necessarily bad. Actually, suffering is highly motivating and often the most efficient source of learning. Coaching can help the patient canalize his motivation into highly constructive considerations and behavior.

Concerning children, we have seen many cases in our daily clinical practice who suffer from chronic pain with psychosomatic components due to poor thriving in their family. The physician has an important job helping the parents to develop as persons, teaching them the basic holding of awareness, respect, care, acknowledgment, and acceptance of their child. Most of the chronic pain and discomfort with children can be improved using the holistic medical toolbox.

## ACKNOWLEDGMENTS

This study was supported by grants from IMK Almene Fond. The quality of life research was approved by the Copenhagen Scientific Ethical Committee under number (KF)V.100.2123/91.

## BIOSKETCHES

**Søren Ventegodt, MD,** is the Director of the Quality of Life Research Center in Copenhagen, Denmark. He is also responsible for a Research Clinic for Holistic Medicine in Copenhagen and is a popular speaker throughout Scandinavia. He has published numerous scientific or popular articles and a number of books on holistic medicine, quality of life, and quality of working life. His most important scientific contributions are the comprehensive SEQOL questionnaire, the very short QoL5 questionnaire, the integrated QOL theory, the holistic process theory, the life mission theory, and the Danish Quality of Life Research Survey, 1991–94 in cooperation with the University Hospital of Copenhagen and the late pediatric professor Bengt Zachau-Christiansen. E-mail: ventegodt@livskvalitet.org. Website: http://www.livskvalitet.org

**Mohammed Morad, MD**, is Specialist in Family Medicine, Lecturer in Family Medicine at the National Institute of Child Health and Human Development, Division of Community Health, Ben Gurion University of the Negev and the Medical Director of a large area clinic in the city of Beer-Sheva. He has publications on Bedouin health, health aspects, spiritual health, and aging in persons with intellectual disability, and is a presenter on topics such as health policy and services for the disadvantaged at national and international conferences. E-mail: morad62@barak-online.net

**Isack Kandel**, **MA, PhD,** is Senior Lecturer at the Faculty of Social Sciences, Department of Behavioral Sciences, the Academic College of Judea and Samaria, Ariel, but during the period 1985–93, Director of the Division for Mental Retardation, Ministry of Social Affairs, Jerusalem, Israel. E-mail: Kandeli@aquanet.co.il

**Joav Merrick, MD, DMSc,** is Professor of Child Health and Human Development affiliated with the Zusman Child Development Center, Division of Pediatrics and Community Health at the Ben Gurion University, Beer-Sheva, Israel; the Medical Director of the Division for Mental Retardation, Ministry of Social Affairs, Jerusalem; and the Founder and Director of the National Institute of Child Health and Human Development. He has numerous publications in the field of child and human development, rehabilitation, intellectual disability, disability, health, welfare, abuse, advocacy, quality of life, and prevention. Dr. Merrick received the Peter Sabroe Child Award for outstanding work on behalf of Danish Children in 1985 and the International LEGO-Prize (“The Children’s Nobel Prize”) for an extraordinary contribution towards improvement in child welfare and well being in 1987. E-mail: jmerrick@internet-zahav.net. Website: www.nichd-israel.com
